# Impact of microbiome-based interventions on gastrointestinal pathogen colonization in the intensive care unit

**DOI:** 10.1177/1756284820939447

**Published:** 2020-07-17

**Authors:** Alexa Choy, Daniel E. Freedberg

**Affiliations:** Department of Medicine, Columbia University Medical Center, New York, NY, USA; Division of Digestive and Liver Diseases, Columbia University Medical Center, New York, NY, USA

**Keywords:** Gut microbiome, microbiome-based intervention, intensive care unit, gastrointestinal colonization, probiotics, prebiotics, synbiotics

## Abstract

In the intensive care unit (ICU), colonization of the gastrointestinal tract by potentially pathogenic bacteria is common and often precedes clinical infection. Though effective in the short term, traditional antibiotic-based decolonization methods may contribute to rising resistance in the long term. Novel therapies instead focus on restoring gut microbiome equilibrium to achieve pathogen colonization resistance. This review summarizes the existing data regarding microbiome-based approaches to gastrointestinal pathogen colonization in ICU patients with a focus on prebiotics, probiotics, and synbiotics.

## Introduction

In the intensive care unit (ICU), colonization of the gastrointestinal tract by potentially pathogenic bacteria is common and often precedes clinical infection. Though effective in the short term, traditional antibiotic-based decolonization methods may contribute to rising resistance in the long term. Novel therapies instead focus on restoring gut microbiome equilibrium to achieve pathogen colonization resistance. The purpose of this review is to summarize the existing data regarding microbiome-based approaches to gastrointestinal pathogen colonization in ICU patients with a focus on prebiotics, probiotics, and synbiotics.

## Gastrointestinal pathogen colonization during critical illness

### Routes of pathogen colonization in the ICU

Admission to the ICU is associated with a dramatic loss of phylogenetic diversity in the gastrointestinal microbiome.^1^ This change in gut microbial composition allows pathogenic organisms, such as *Clostridium difficile* (more recently reclassified as *Clostridioides difficile*^2^), *Pseudomonas aeruginosa, Candida* species, vancomycin-resistant enterococci (VRE), and other multidrug resistant organisms (MDRO) to proliferate within and colonize the gut.^1,3–6^

Critical illness itself can facilitate gastrointestinal pathogen colonization. Sepsis, one of the most common conditions in the ICU, is characterized by a dysregulated host response to infection that appears to alter gut microbial composition for the worse.^7,8^ Sepsis disrupts gut integrity *via* endogenous catecholamine production,^9^ gut hypoperfusion,^10^ degradation of the intestinal mucus layer,^11^ and decreased bile salt production.^12^ This in turn disrupts the intestinal microenvironment, allowing pathogenic organisms to dominate and existing bacteria to become more virulent.^1,6^

Many of the interventions in the ICU also impact susceptibility to enteric colonization. Broad-spectrum antibiotics are used in 70% of medical ICU patients^13^ and are an important risk factor for pathogen colonization.^14–18^ Antibiotic exposure depletes the microbiome of many endogenous species leaving the gut highly vulnerable to colonization by antibiotic-resistant pathogens.^19^ Antibiotics can also facilitate the proliferation of resistant bacteria already present in the gut by eliminating antibiotic-sensitive competitors within the same species. This increases the relative abundance of antibiotic-resistant bacteria in the gut and ultimately leads to increased dissemination of these strains into the ICU environment.^20^ Transmission of resistant bacteria between patients can then occur *via* contact with healthcare workers, adjacent patients, or contaminated objects.^21^ Antibiotics therefore impact colonization pressure at the ward level,^22^ increasing colonization and infection risk in patients who do not themselves receive antibiotics.^23^ Other common ICU interventions, such as proton pump inhibitors,^24^ immunosuppressive agents,^25^ and enteral feeding (or lack of feeding)^26^ also alter the gut microbiome and influence colonization of the digestive tract.

### Implications of gastrointestinal pathogen colonization

Around 4–11% of all patients in the ICU have guts that are colonized with methicillin-resistant *Staphylococcus aureus* (MRSA), VRE, or MDR Gram-negative bacteria at the time of ICU admission;^27–31^ among patients who are free of MDROs on admission, an additional 12–14% become colonized during their ICU stay.^32^ Gastrointestinal colonization with MDROs increases risk for subsequent clinical infection with the colonizing organisms as much as 10-fold.^31,33–35^ Subsequent mortality rates are high with MDR infection causing up to 9 deaths for every 100 patients admitted to the ICU.^36^

Colonization by *Candida* species is also very common and can occur in up to 80% of critically ill patients after 1 week in the ICU.^37^ Common species include *C. albicans, C. glabrata, C. parapsilosis, C. krusei*, and *C. tropicalis*.^38^ Colonization is a well-established risk factor for subsequent *Candida* infection,^39,40^ leading to the development of the *Candida* colonization index as an early warning tool for invasive candidiasis.^38,41^ Although the proportion of colonized ICU patients who later develop invasive candidiasis is low,^42^ associated mortality from invasive infection ranges from 5% to 71%.^43–46^

Gastrointestinal colonization can also increase risk for extra-intestinal infections. When patients shed gastrointestinal bacteria into the ICU environment *via* feces, subsequent inoculation back into the patient *via* contaminated intravenous or urinary catheter insertion can occur.^47^ Other more direct routes of infection can also occur, such as aspiration of gastric contents or bacterial translocation across edematous bowel. While it is unknown how frequently such events happen, an increasingly large number of studies support the idea that most new healthcare-associated infections do not come from other patients but instead from colonizing bacteria present within the patient’s own gut at the time of hospital admission.^48,49^

Interventions aimed at the prevention or eradication of gastrointestinal pathogens therefore have the potential to prevent clinical infection.

## Traditional approach to pathogen colonization

Selective digestive decontamination (SDD) has shown effectiveness for gut decolonization since the 1980s.^50^ SDD relies on prophylactic administration of oropharyngeal and enteral antimicrobials throughout the ICU stay coupled with a parenteral antibiotic within 4 days of ICU admission.^51^ SDD antibiotics ideally target potential pathogens such as aerobic Gram-negative rods, *Staphylococcus aureus*, and yeasts while trying to minimize perturbation to endogenous, anaerobic flora. High-quality randomized trials show that SDD is effective in reducing ICU-acquired infections by pathogenic gut colonizers and may even provide mortality benefit,^52–54^ though some major studies have been negative.^55^ Long-term studies have shown reduction in the unit-level use of antibiotics, underlining the potential benefit of SDD in ICU settings.^51^

Despite relatively strong supporting evidence, SDD has faced concern that the selective pressure of antibiotics will lead to the emergence of new resistance. Rebound increases in resistant pathogens after SDD have been demonstrated in a handful of studies,^56,57^ yet there is surprisingly little evidence that SDD leads to long-term MDRO colonization and infection, as one might fear it would.^58^ A 21-year longitudinal study on the long-term ecological effects of SDD found no significant increase in the incidence rates of resistant microbes at the ICU level despite an overall increase in antimicrobial resistance at the hospital level.^59^ This study was conducted in a region with low rates of resistance, and SDD trials have been centered at a few large European institutions making their results less generalizable. Perhaps the most candid assessment of SDD is that clinicians are reluctant to use antibiotics to combat a fundamentally antibiotic-related problem.

## Pre-, pro-, and synbiotics for pathogen colonization

Given the lack of widespread adoption of SDD, alternative approaches to gastrointestinal colonization are needed for the ICU. Supplementation with probiotics, in tandem with or separately from a prebiotic, has been hypothesized as a safe, cost-effective approach to colonization resistance. We review in detail the current evidence regarding the impact of prebiotics, probiotics, and synbiotics on gastrointestinal colonization and clinical outcomes in ICU patients.

### Prebiotics

Prebiotics are non-digestible dietary components, such as oligosaccharides, fiber, and inulin, that selectively promote the proliferation of commensal gut microbiota.^60^ In theory, prebiotic supplementation could enhance the growth and protective effects of beneficial endogenous flora in critically ill patients and confer a transitive benefit to the host through colonization resistance. This benefit could be because the modified flora directly competes with and crowds out pathogens, or because prebiotic fiber is metabolized into short-chain fatty acids (SCFAs) which have direct benefits, or from other mechanisms. There is also evidence in animal models that prebiotics can have microbiome-independent effects that modulate the host inflammatory response directly. A recent study found that exposure to prebiotics alters the response to pathogen-induced kinase activation in intestinal epithelial cells and dampens the inflammatory response to lipopolysaccharide *in vivo* without altering gut microbiota.^61^ Other studies have shown that non-digestible oligosaccharides influence B-cell responses and macrophage markers in mice with and without a microbiome.^62,63^

A handful of studies have investigated prebiotics in the ICU. O’Keefe *et al*. examined the short-term clinical tolerance and fecal SCFA concentrations in response to progressive fiber supplementation in 13 ICU patients.^64^ After fiber supplementation, there was a dramatic increase in Firmicutes and other SCFA producers and an increase in their metabolites, acetate, propionate, and butyrate.^64^ Our own retrospective study of 129 ICU patients demonstrated a similar increase in SCFA producers with higher amounts of fiber.^65^ These findings, assuming SCFAs are indeed beneficial,^66^ support the value of fiber and other prebiotics in maintaining gut microbial homeostasis.^64^ However, other trials, including a study of oligofructose/inulin in ICU patients receiving enteral nutrition, have shown no impact on the microbiome.^67^

There is also no compelling evidence that prebiotics can impact clinical outcomes. A prospective, single-blind randomized trial with 237 ICU patients investigated the impact of a high-protein formula enriched with arginine, fiber, and antioxidants on the rates of ICU-associated infection, length of stay, and mortality.^68^ While no significant differences in mortality were observed, the incidence of catheter-related sepsis was significantly lower in the intervention group (0.4 episodes/1000 ICU days *versus* control 5.5 episodes/1000 ICU days).^68^ Another study (30 patients) comparing early enteral nutrition with prebiotic fiber supplementation in ICU patients with severe pancreatitis *versus* standard enteral feeds found a reduction in hospital length of stay (10 days *versus* 15 days) and lower rates of complications, including multiorgan failure, sepsis, and death.^69^

The impact of prebiotics on gastrointestinal pathogen colonization has been examined in a small number of studies in non-ICU patients. A recent study profiled the microbiome of 87 children treated with azithromycin with or without lactulose. Patients in the azithromycin-only group had a statistically significant increase in the relative abundance of pathogenic bacteria such as *Streptococcus* that was not demonstrated in the prebiotic supplementation group. The prebiotic group also had higher fecal concentrations of protective *Lactobacillus* species.^70^

As these studies suggest, prebiotic trials have been heterogeneous both in terms of the interventions tested and in terms of trial outcomes. Overall, data supporting prebiotics for gastrointestinal pathogen colonization in ICU patients are sparse.

### Probiotics

Probiotics are live, ingestible microorganisms that can confer health benefits on to their host.^71^ The mechanisms by which these symbionts may deter pathogen colonization include competition, reduction of gut pH, enhancement of innate and adaptive immunity, and production of antimicrobial substances.^71,72^ Several systematic reviews and meta-analyses have evaluated the benefits of probiotics in critically ill patients.^73–77^ Probiotic administration has been associated with several favorable infection-related outcomes, including reduced incidence of overall infections,^74^ and in ICU-acquired^76^ and ventilator-associated pneumonia.^74^

At least 5 randomized-controlled studies with 48–208 patients have investigated the effects of *Lactobacillus*-based probiotics on gut colonization in adult ICU patients. The first study investigated the impact of a probiotic drink containing 5 × 10^7^ colony-forming units (CFU) per ml of *Lactobacillus plantarum*, on gastric colonization *via* nasogastric aspirate at days 1, 4, and 8 of ICU admission. The study enrolled 103 patients and also investigated intestinal permeability, endotoxin exposure, inflammatory marker levels, and overall sepsis morbidity and mortality. No difference was detected in pathogen colonization related to the intervention. The probiotic group did exhibit significantly lower interleukin-6 levels at day 15 compared with controls, however clinical outcomes, i.e. sepsis complications and mortality, were unaffected.^78^

Another study investigated the effect of *L. plantarum* 299v on *Clostridioides difficile* colonization in 48 ICU non-colonized patients.^79^ Around 19% of control patients had positive rectal swabs for *C. difficile* by ICU discharge compared with zero *C. difficile-*positive patients in the probiotic treatment group. They concluded that probiotic supplementation could reduce *C. difficile* colonization rates in the ICU but that interpretation of these results should be taken with caution given the study’s small sample size and premature termination due to loss of funding.^79^ A more recent study investigating the feasibility of a *L. casei* drink found a nonsignificant trend towards lower rates of antibiotic-associated diarrhea and *C. difficile* among ICU patients receiving antibiotics compared with a contemporary control.^80^ The efficacy of other probiotic strains for the treatment and prevention of *C. difficile*, including *Saccromyces boulardii*, has been reviewed elsewhere.^81–84^ Systematic reviews and meta-analyses suggest (with moderate certainty) that probiotics are effective for preventing *C. difficile*-associated diarrhea, though few studies have specifically studied *C. difficile* prevention using probiotics in the ICU.^84^ In appropriately selected high-risk populations, probiotics probably do decrease *C. difficile* incidence, but the effect size is likely small and the optimal probiotic is unknown.

Three studies investigated the effects of probiotics primarily containing *L. rhamnosus*. A study conducted in a single ICU randomized 208 adults with a unit stay longer than 48 h to either *L. casei rhamnosus* (10^9^ CFU) or placebo *via* nasogastric tube from day 3 after admission until discharge or death. The primary outcome was time to *Pseudomonas aeruginosa* acquisition as measured in weekly gastric aspirates. Although there was no significant difference in median time before gastric acquisition (16 days in the treatment group *versus* 30 days in placebo), the probiotic group was found to have a significant delay in respiratory colonization (50 days *versus* 11 days).^85^

Another study investigated the impact of a probiotic capsule containing primarily *L. rhamnosus GG, L. casei, L acidophilus*, and *Bifidobacterium bifidum* on mortality, infection, and nasal/gastrointestinal colonization in 167 ICU patients mechanically ventilated for longer than 48 h.^86^ No significant differences in mortality, colonization, or hospital-acquired infections were found. Catheter-related bloodstream infections were lower in the probiotic group compared with placebo (1.8% of catheter days in the treatment group *versus* 6.8% control). The authors also conducted a preplanned subgroup analysis of 101 patients who met the criteria for severe sepsis. Severely septic patients treated with probiotics had a threefold reduction in 28-day mortality compared with those in the placebo group. However, an almost equally increased risk for 90-day death was found in non-severely septic patients receiving probiotics. Whether or why the physiology of sepsis might modify the effects of a *Lactobacillus*- and *Bifidobacterium*-containing probiotic is unclear.^86^

A pilot study of 70 ICU patients tested *L. rhamnosus* for prevention of colonization with carbapenem-resistant Enterobacteriaceae (CPE), VRE, extended spectrum β-lactamase producing Enterobacteriaceae (ESBL-E), *Pseudomonas*, or *C. difficile*. Colonization was defined as negative stool or rectal culture results at enrollment with subsequent positive culture results on day 3 and/or at study exit. No difference was found in the colonization rates between the probiotic and standard of care groups (10% of intervention group *versus* 15% standard of care), and none of the treatment group patients lost colonization with VRE, *P. aeruginosa*, or *C. difficile* by the specified time points.^87^

These probiotic studies have some promising results but do not demonstrate a consistent impact on gut pathogen colonization in the ICU. There are also several relevant non-ICU studies that have tested probiotics for the prevention of colonization. A small, single-center, double-blind randomized controlled trial (RCT) was conducted to determine whether *L. rhamnosus GG*-containing yogurt consumption could eradicate VRE carriage in renal ward patients with VRE-positive swabs on admission. All treatment-group patients (*n* = 11) who completed the study had cleared VRE at the 4-week endpoint *versus* 1 out of 12 in the control group, and 8 out of 11 remained VRE negative at 1 month after study completion. All remaining patients in the control group who had failed to clear VRE at the 4-week time point were then crossed over to receive the probiotic-containing yogurt and had cleared VRE by 8 weeks. Of note, there was a greater antibiotic usage in the probiotic group during the study (10/14 *versus* 5/13), and 2 patients in the probiotic group received linezolid (to which VRE is susceptible).^88^

A Swedish randomized trial of 80 patients using a mixture of 8 bacterial strains (primarily *Lactobacillus* and *Bifidobacterium*) found that the probiotic was not superior to placebo at eradication therapy in adult outpatients intestinally colonized with ESBL-E for at least 3 months. There was a nonsignificant trend towards successful decolonization in the probiotic group (13% in probiotic group, 5% in control), with limited power.^89^ Other non-ICU studies have failed to demonstrate a significant impact of probiotics on gastrointestinal colonization by highly resistant organisms. A study of 530 elderly residents in a long-term care facility found no reduction in fecal norfloxacin-resistant *Escherichia coli* in patients treated with the probiotic product *E. coli* strain Nissle 1917 (Mutaflor).^90^ Similarly, probiotics did not reduce gastrointestinal carriage rates of ampicillin-resistant *Enterococcus faecium*^91^ or VRE^92^ in patients admitted to non-ICU wards with a high prevalence of these resistant organisms.

Like the ICU studies, these outpatient studies have some positive results but do not provide sufficient evidence to support probiotics as an effective method for preventing or eradicating gastrointestinal pathogen colonization. Ultimately the differences in methodology, especially in the composition of the probiotic and the operationalization of colonization, make it hard to draw firm conclusions.

### Synbiotics

Synbiotics are supplements which contain both probiotic organisms and their prebiotic nutritional substrates as a method to facilitate their survival in the gastrointestinal tract. Such an approach seems logical, but the packaging and drug delivery issues are non-trivial. As a result, many synbiotic RCTs have been sponsored by the synbiotic manufacturer.

Jain *et al*. conducted a 1:1 RCT that compared the incidence and nature of gastric colonization in patients receiving a synbiotic supplement containing *Lactobacillus acidophilus, Bifidobacterium lactis, Streptococcus thermophiles*, and *L. bulgaricus* with oligofructose to those receiving placebo.^93^ Patients in the treatment group had significantly fewer gastric aspirates growing multiple strains of bacteria or fungi (9 patients synbiotic *versus* 18 placebo) as well as a lower incidence of colonization by prespecified potentially pathogenic organisms such as *E. coli* and *Enterococcus faecalis* (10 *versus* 18, respectively). Synbiotic supplementation was not associated with improved sepsis outcomes although the study was small.^93^

Salomão *et al*. studied the effect of a synbiotic containing *L. bulgaricus* and *L. rhamnosus* suspended in a fructo-oligosaccharide prebiotic mixture on the eradication of MDR Gram-negative bacilli colonization in adult patients. Although the authors considered a heterogeneous group of patients, 42% of patients in the experimental arm and 32% of patients in the control arm were in an ICU. No significant differences in decolonization were found between the 2 groups (11 placebo *versus* 8 synbiotic). Systemic antibiotics were used frequently in both groups, which may have influenced both probiotic and pathogenic viability.^94^

Studies investigating the effects of synbiotics have also been conducted in ICU subpopulations, including liver transplant recipients,^95^ trauma patients,^96^ patients with acute pancreatitis,^97^ and patients undergoing major abdominal surgery.^98^ While these studies did not specifically evaluate outcomes related to gastrointestinal colonization, synbiotics were associated with lower incidence of pneumonia, postoperative infections,^95,96,98^ and sepsis complications during pancreatitis.^97^

## Novel approaches to pathogen colonization

### Antimicrobial peptides

There are several novel microbiome-based therapies, not yet tested in clinical trials, that deserve mention. An interesting approach involves harnessing the ability of some bacteria to inhibit the growth of closely related organisms through the production of antimicrobial peptides called bacteriocins.^99^ Bacteriocins are primarily produced by members of the Firmicutes phylum, including *Lactobacillus, Staphylococcus, Acetobacterium*, and *Streptococcus*.^100^ These antimicrobials are active against a narrow spectrum of closely related competitors,^99^ so they are an attractive, highly specific therapeutic target.

Several animal model studies have investigated the ability of bacteriocins to inhibit colonization of gastrointestinal pathogens. *Enterococcus faecalis* conjugated with a bacteriocin-expressing plasmid pPD1 effectively outcompetes strains without the plasmid and inhibits growth of multidrug-resistant enterococci.^101^ Similar results have been found in the ability of bacteriocin-producing bacteria to inhibit various *Streptococcus* species,^102^ *Salmonella enteritidis*,^103^ *Listeria monocytogenes*,^104^ and *Clostridioides difficile.*^105–107^

A related approach was taken by Kim *et al*. who investigated the effectiveness of a four-strained consortium of commensal bacteria (*Clostridium bolteae, Blautia producta, Bacteroides sartorii, and Parabacteroides distasonis*) at providing resistance to VRE, a common ICU pathogen, in mice.^108^ The strain of *B. producta* studied, BPscsk, was found to secrete a lanthionine-containing bacteriocin, or lantibiotic, that inhibited VRE colonization. The authors demonstrated that abundance of this lantibiotic in patient fecal samples was inversely proportional to the relative abundance of *E. faecium*.^108^ These results suggest that lantibiotics and other bacteriocins can improve colonization resistance, although all the current data come from studies performed in animals. Novel therapies could either selectively promote the growth of bacteriocin-producing bacteria or synthetically reproduce the bacteriocins themselves. The idea of encouraging the growth of benign bacteriocin-producing bacteria has appeal, but a concern is that such organisms might themselves acquire pathogenic traits (e.g. by picking up plasmids containing antibiotic resistance).

### Bacteriophage therapy

Bacteriophages are viruses that specifically infect bacteria.^109^ Their ability to lyse bacteria at the site of infection can be harnessed as a microbiome-targeted alternative to antibiotics.^110^ One proposed advantage of bacteriophage therapy is its specificity for a particular bacterial target, resulting in minimal disruptions to other organisms in the gut microbiome.^110^ The efficacy of bacteriophage therapy in targeting gastrointestinal pathogens has been investigated in a limited number of studies. Nale *et al.* demonstrated that delivery of optimized bacteriophage combinations significantly reduced *Clostridioides difficile* colonization *in vitro* and *in vivo.*^109^ Sterile filtrates from donor stool have also been shown to improve symptoms in patients with *C. difficile* infection (CDI), suggesting that bacteriophages and other nonbacterial components of the microbiome can influence colonization.^111^

Several barriers preclude widespread use of bacteriophage therapy for gastrointestinal pathogen decolonization, including a limited understanding of the cascading effects on other organisms in the microbiome and a lack of safety and efficacy data in humans.^112^ There are currently no high-quality data supporting the use of bacteriophage therapy for gastrointestinal pathogen colonization in the ICU.

### Competitive inhibition of pathogenic strains

Another interesting decolonization approach harnesses the use of nonpathogenic strains to outcompete their pathogenic counterparts for space and nutrients in the gastrointestinal microbiome. This is best demonstrated in the *Clostridioides difficile* literature. A phase II RCT conducted in 44 centers across the USA, Canada, and Europe examined the safety, fecal colonization, recurrence rate, and optimal dosing schedule of nontoxigenic *C. difficile* strain M3 (NTCD-M3) spores for prevention of recurrent CDI.^113^ Patients who received NTCD-M3 spores experienced significantly lower rates of CDI recurrence (11% in NTCD-M3 patients *versus* 30% of placebo patients); these rates were even lower in patients who were colonized with NTCD-M3.^113^ Although this study demonstrated few safety concerns among patients in the intervention arm, *in vitro* studies have shown that NTCD strains can acquire the toxin A and B pathogenicity locus from toxigenic strains.^114^ While this has not been demonstrated *in vivo*, phase III studies are necessary to confirm safety and efficacy.

### Fecal microbiota transplantation

Fecal microbiota transplantation (FMT) restores gut biodiversity by reintroducing normal gut flora from healthy donors.^115^ Among patients with recurrent CDI*s*, FMT has also been associated with a reduction of antimicrobial resistance genes in stool microbiota.^116^ The use of FMT for the treatment of refractory CDI is well established and has been incorporated into guidelines as a treatment option in recurrent CDI.^117^

The success of FMT for CDI led to investigation into its effects on other intestinal pathogens. The thinking is that if FMT, an intervention that comes as close as possible to ‘resetting’ the gut microbiome, does not effectively prevent pathogen colonization, then nothing will. Woodworth *et al*. recently reviewed 10 FMT studies and case reports with antibiotic-resistant organism decolonization as a primary endpoint and 7 with decolonization as a secondary endpoint. They concluded that while the evidence supports FMT as a method for eradicating colonization by various types of multidrug-resistant bacteria, these studies all have serious limitations including a lack of true controls and long-term safety data.^118^ More recently, Huttner *et al*. randomized 39 adults colonized with extended ESBL-E and/or CPE to either no intervention or a 5-day course of antibiotics followed by FMT. Although the intervention group experienced a slightly lower rate of ESBL-E/CPE colonization, the results did not achieve statistical significance and the conclusions were limited overall by the study’s small sample size.^119^

Only a small handful of cases have been reported detailing the use of FMT among ICU populations.^120^ Also, while FMT is reasonably safe, high-profile cases of bacteremia have been reported, in one case leading to death.^121^ Currently, the jury is out on FMT in the ICU. Well-designed studies are needed but may be difficult to implement.

## Nutrition in the ICU

Pre-, pro-, and synbiotic ICU data come from single-center studies usually with <100 patients whereas ICU nutrition trials have been multicenter and powered with thousands of patients. Nutrition trials have not focused on MDRO colonization or the microbiome but rather have investigated the optimal timing, route, and nutrient composition for feeding ICU patients. Early enteral feeding within 24–48 h of ICU admission has been the favored approach and is supported by guidelines.^122^ This recommendation is based on meta-analyses of smaller studies, and the largest and highest-quality studies have failed to demonstrate a clear clinical difference between early *versus* late feeding. Though enteral nutrition has been associated with a lower risk of infections compared with parenteral nutrition, benefits in mortality or other clinical outcomes have also not been consistently demonstrated.^123,124^ Finally, optimal caloric intake in critically ill patients has also been a subject of debate. While some studies have shown a mortality benefit among patients with a higher daily calorie intake,^125^ others have demonstrated no significant differences in clinical outcomes among patients permissively underfed^126^ or receiving smaller, trophic feeds compared with those receiving full feeding.^127^ Implementation of evidenced-based guidelines has been associated with earlier initiation^128^ and longer duration of nutrition,^125^ however the impact on clinical outcomes has not been consistently demonstrated.

If ICU trials of nutrition have been null or (at best) unconvincing, does that imply pre- or probiotics will be unable to prevent MDRO colonization in the ICU? None of the large ICU nutrition trials were designed to evaluate colonization specifically, so such a conclusion would be premature. The nutrition literature is a cautionary tale for those seeking to develop microbiome-based interventions, but does not mean such interventions are hopeless.

## Conclusion

Gastrointestinal colonization by pathogenic, multidrug-resistant bacteria is common among ICU patients and is a precursor to life-threatening infections and multiorgan dysfunction. Microbiome-based therapies offer an attractive alternative to traditional, antibiotic-centric decontamination efforts by enhancing the proliferation of beneficial symbionts and (hopefully) restoring gut microbial equilibrium. The existing evidence for such therapies is encouraging yet quite inconclusive as to whether pre-, pro-, or synbiotics can ameliorate pathogen colonization in the ICU (see summary Table 1). Future studies should state exactly what is being studied, and ideally, why. Such studies must predesignate the outcomes of interest. If MDRO pathogens are the targets, which organisms and precisely how will they be assessed? Is the intervention being tested to eradicate gut colonization that was already present at the time of ICU admission, or to prevent the acquisition of new MDROs during hospitalization? Culture remains the clinical gold standard for diagnosis of almost all important nosocomial pathogens; future studies may want to include culture for predesignated MDROs, as opposed to only sequencing results, as a way of assessing the effectiveness of interventions. To be convincing, studies of microbiome-based interventions will need to be relatively large, blinded, randomized, and appropriately controlled. Microbiome-based interventions have a bright future in the ICU but much work needs to be done before such interventions enter the clinical realm.

**Table 1.** Prebiotics, probiotics, and synbiotics for MDRO colonization.

Intervention type	Common intervention components	Strengths	Limitations	RCTs with ⩾20 patients discussed in the review	GRADE evidence for MDRO colonization
PREBIOTICS	Fiber, inulin, oligosaccharides	• No living bacteria and therefore likely safe • Ease of administration • Inexpensive	• May require dilution in relatively large volume of water to be given at high dose • May be ineffective if commensals are completely depleted prior to the intervention	Majid^67 $^ Caparrós^68 * ‡^ Karakan^69 $^	Weak
PROBIOTICS	*Lactobacillus rhamnosus, L. casei, L. plantarum, Bifidobacterium bifidum*	• Appear safe in the ICU • Fewer side effects and interactions than conventional pharmaceuticals • Enhance natural defenses	• Variable composition • Lack of standardized dosing • Susceptibility to antibiotics/lack of bacterial viability • Possibility of harm in select populations, including immunocompromised	McNaught^78 ‡^ Forestier^85 * ‡^ Klarin^79 * ‡^ Barraud^86 $^ Kwon^87^	Weak
SYNBIOTICS	Combinations of the prebiotics and probiotics above	• Improved survival of probiotic bacteria	• Packaging/drug delivery issues • Virtually all data to date are industry-sponsored	Jain^93 $^ Salomão^94 $^ Rayes^95 $^ Spindler-Vesel^96 $^ Oláh^97 $^ Rayes^98^	Weak

* Industry sponsored.

$ Double-blinded.

‡ Single-blinded.

GRADE, Grading of Recommendations, Assessment, Development and Evaluations; ICU, intensive care unit; MDRO, multidrug resistant organisms; RCT, randomized control trial.
